# Supplementary material for: Liquid Biopsy as a Means of Assessing Prognosis and Identifying Novel Risk Factors in Multiple Myeloma
Source: Int J Mol Sci. 2025 Sep 1;26(17):8505. doi: 10.3390/ijms26178505 (PMC12429773; doi:10.3390/ijms26178505)
Supplement: Supplementary file 1 [file ijms-26-08505-s001.zip › Supplementary Figures S1-S3.pdf]

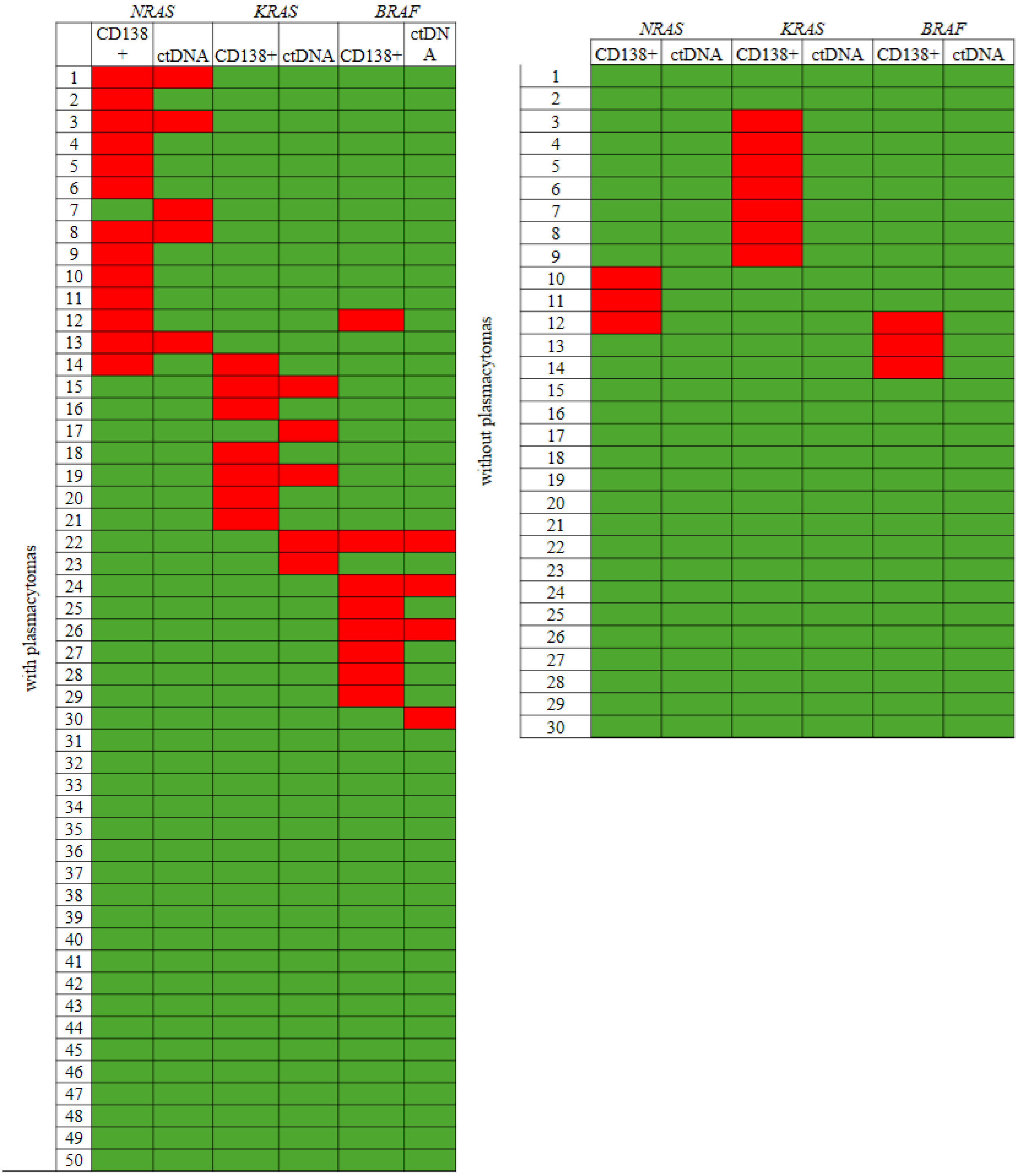

NRAS
KRAS
BRAF
CD138+
ctDNA
CD138+
ctDNA
CD138+
ctDNA
with plasmacytomas
1 2 3 4 5 6 7 8 9 10 11 12 13 14 15 16 17 18 19 20 21 22 23 24 25 26 27 28 29 30 31 32 33 34 35 36 37 38 39 40 41 42 43 44 45 46 47 48 49 50
NRAS
KRAS
BRAF
CD138+
ctDNA
CD138+
ctDNA
CD138+
ctDNA
without plasmacytomas
1 2 3 4 5 6 7 8 9 10 11 12 13 14 15 16 17 18 19 20 21 22 23 24 25 26 27 28 29 30

Figure S1: Mutations in the RAS-ERK cascade genes in MM patients with and without plasmacytoma. Red indicates the presence of a mutation in the gene, and green indicates the absence.

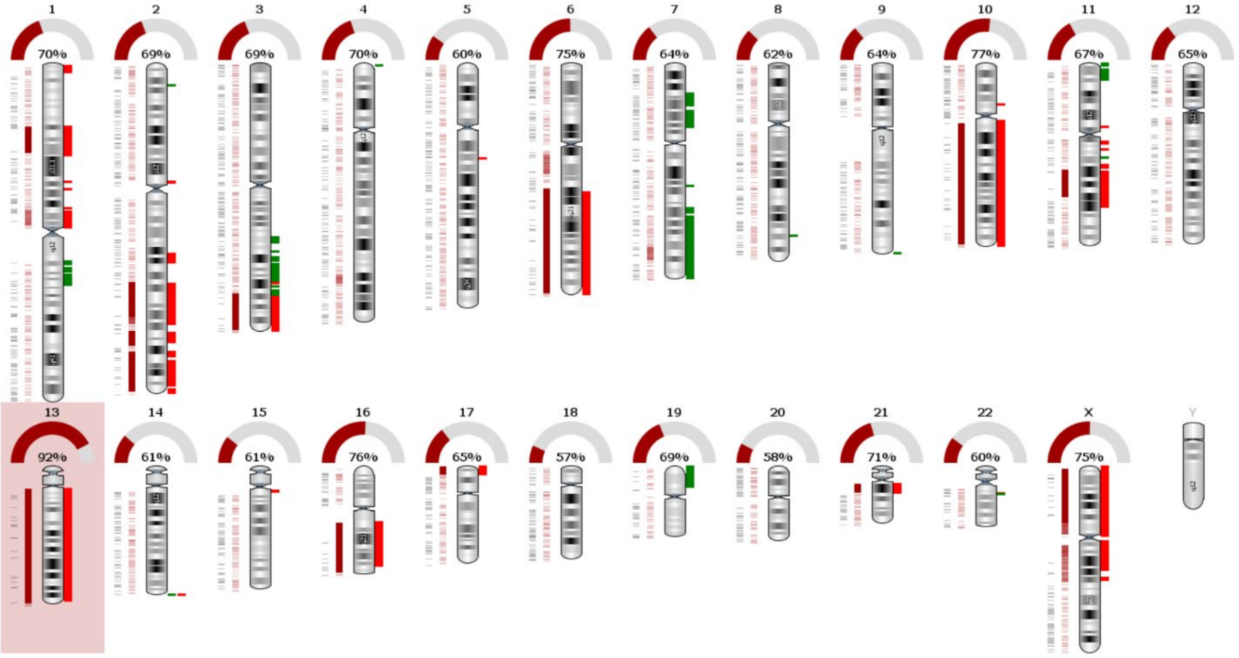

Figure S2. Molecular karyotype of an extramedullary plasmacytoma in patient №12. Duplications are marked in green, deletions are marked in scarlet, loss of heterozygosity are marked in red.

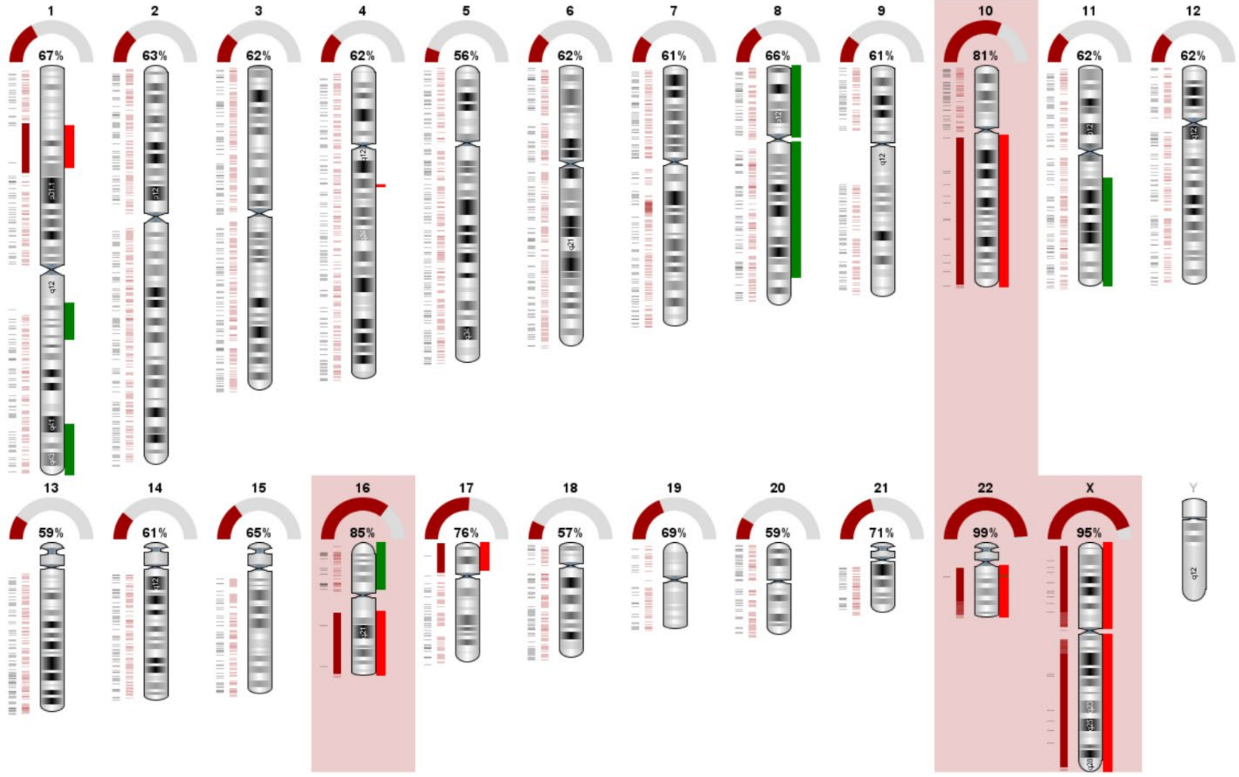

Figure S3. Molecular karyotype of an extramedullary plasmacytoma in patient №24. Duplications are marked in green, deletions are marked in scarlet, loss of heterozygosity are marked in red.
